# Unveiling a Novel Antidote for Deoxynivalenol Contamination: Isolation, Identification, Whole Genome Analysis and In Vivo Safety Evaluation of *Lactobacillus rhamnosus* MY-1

**DOI:** 10.3390/foods13132057

**Published:** 2024-06-28

**Authors:** Jie Yao, Songbiao Chen, Yijia Li, Chengshui Liao, Ke Shang, Rongxian Guo, Jian Chen, Lei Wang, Xiaojing Xia, Zuhua Yu, Ke Ding

**Affiliations:** 1 Laboratory of Functional Microbiology and Animal Health, Henan University of Science and Technology, Luoyang 471023, China; yj18403798226@163.com (J.Y.); chensongbiao@126.com (S.C.); 13462205724@163.com (Y.L.); liaochengshui33@163.com (C.L.); shangke0624@163.com (K.S.); guorongxian520@163.com (R.G.); chenillejian@163.com (J.C.); 2 Luoyang Key Laboratory of Live Carrier Biomaterial and Animal Disease Prevention and Control, Henan University of Science and Technology, Luoyang 471003, China; 3 The Key Lab of Animal Disease and Public Health, Henan University of Science and Technology, Luoyang 471023, China; 4 Ministry of Education Key Laboratory for Animal Pathogens and Biosafety, Zhengzhou 450000, China; wlei@163.com (L.W.); quik500@163.com (X.X.); 5 College of Animal Science and Veterinary Medicine, Henan Institute of Science and Technology, Xinxiang 453003, China

**Keywords:** deoxynivalenol, *Lactobacillus rhamnosus*, biodegradation, safety test, whole-genome analysis

## Abstract

Deoxynivalenol (DON) is a global contaminant found in crop residues, grains, feed, and animal and human food. Biodegradation is currently the best solution for addressing DON pollution. However, efficient detoxification bacteria or enzymes that can be applied in complex matrices are lacking. The aim of this study was to isolate a DON-detoxifying probiotic strain with a high degradation rate, a good safety profile, and a clear genetic background. One hundred and eight bacterial strains were isolated from 300 samples collected from a school farm and surrounding livestock farms. A new DON-degrading strain, *Lactobacillus rhamnosus* MY-1 (*L. rhamnosus* MY-1), with a degradation rate of 93.34% after 48 h and a comprehensive degradation method, was identified. Then, MY-1 at a concentration of 1 × 10^8^ CFU/mL was administered to mice in a chronic intoxication experiment for 28 days. The experimental group showed significantly higher weight gain and exhibited good production performance compared to the control group. The length of the ileal villi in the experimental group was significantly longer than that in the control group. The expression of pro-inflammatory cytokines decreased, while the expression of anti-inflammatory factors increased in the experimental group. Whole-genome analysis revealed that most of the MY-1 genes were involved in carbohydrate metabolism and membrane transport, with a cluster of secondary metabolite genes encoding antimicrobial properties. In summary, this study successfully identified a *Lactobacillus* strain with good safety performance, high DON degradation efficiency, and a clear genetic background, providing a new approach for the treatment of DON contamination.

## 1. Introduction

Mycotoxins, as secondary metabolites, are serious contaminants of food and feed and a threat to human health via the food chain [1]. Deoxynivalenol (DON), also known as vomitoxin, is one of the most common mycotoxins produced by the genus *Fusarium* (*Fusarium* spp.) [2]. Currently, DON contamination is a global problem, and is widely found in crop residues, grains, feeds, and foods [3]. DON poisoning leads to major health issues in both humans and animals, stemming from neurotoxicity [4], cytotoxicity [5], immunotoxicity [6] and enterotoxicity [7].

The degradation of DON primarily encompasses three methodologies: physical, chemical, and biological [8]. The physical and chemical removal of DON often results in incomplete detoxification, low efficiency, high cost, harsh conditions, and affects the nutrition and taste of feeds. Biodegradation, however, has the advantages of a high degradation rate and low cost, while not affecting the nutritional value of feeds, and has become an emerging method for DON detoxification [9]. Biodetoxification includes microbial and bioenzymatic methods, where the degradation of DON in probiotics is primarily attributed to three mechanisms: antagonistic inhibition, bacterial adsorption and bioenzyme degradation [10,11]. Antagonistic bacteria grow competitively with toxigenic strains to partially inhibit the growth of toxigenic strains. For example, *Bacillus amylolytica* can secrete lipopeptide antibiotics while growing, inhibiting the growth of *Fusarium graminea* by more than 60% [12]. Bacterial adsorption mainly uses bacterial cell wall proteins to remove toxins, such as mannose, peptidoglycan and small molecular proteins. Chlebicz et al. reported that the toxin content of DON decreased by 19–39% and 22–43% after 24 h of adsorption in a bacterial solution of *Lactobacillus* sp. and *Saccharomyces cerevisiae*, respectively [13]. Enzyme degradation involves the utilization of extracellular and intracellular enzymes secreted by bacteria to convert DON into non-toxic byproducts with lower molecular weights. Jing Zhang et al. reported that the dehydrogenase DDH produced by *Pelagibacterium halotolerans* ANSP101 can convert DON into 3-epi-DON [14]. Previous studies on the removal of DON by lactic acid bacteria showed that most lactic acid bacteria removed DON by adsorption [15,16,17]. However, there are a few studies on the combined effect of adsorption and degradation by lactic acid bacteria. Li et al. demonstrated that probiotics can mitigate the effects of mycotoxins through a combination of bacterial adsorption and biodegradation [18]. Therefore, identifying a strain of bacteria that can detoxify DON by adsorption and biodegradation has great practical potential.

In this study, we screened microorganisms capable of degrading DON from moldy straw. We successfully isolated a strain demonstrating efficient DON degradation, identified as *Lactobacillus rhamnosus* MY-1, through 16S rRNA analysis. Furthermore, mouse safety experiments and whole-genome analysis of the MY-1 strain have laid the foundation for developing the microbial detoxification of DON.

## 2. Materials and Methods

### 2.1. Isolation and Identification of Bacterial Strains

Three hundred samples of soil, silage and moldy straw were obtained from the Henan University of Science and Technology. One gram of each sample was mixed with 10 mL of sterile water and the suspension was shaken. Then, an inoculation ring dipped in the sample suspension was used to inoculate LB (lysogeny broth) solid medium and MRS (DeMan, Rogosa and Sharpe) solid medium containing 1 μg/mL DON, and placed in an incubator (aerobic and anaerobic environments) at a constant temperature of 37 °C for cultivation over 24 h. Then, single colonies with different morphologies were picked from the media using the inoculating ring for pure cultivation. The strains obtained from the initial screening were activated, inoculated into a LB liquid medium or MRS liquid medium containing 1 μg/mL of DON and re-screened for vomitoxin-degrading bacteria with high degradation capacity.

The culture colonies were screened on MRS agar plates several times, and the morphological characteristics of individual colonies were observed after purification. Individual colonies were selected using an MRS solid medium, and the strain’s morphological features were examined by Gram staining under a microscope.

### 2.2. 16S rRNA Sequencing of MY-1 Strain

Total DNA was extracted from the MY-1 strain using a bacterial genome kit (TIANGEN Biochemical Technology, Beijing, China), following the manufacturer’s recommendations. After extraction, PCR amplification was performed using the universal 16S primers F (5′-AGAGTTTGATCCTGGCTCAC-3′) and R (5′-AAGGAGGTGATCCA-3′), following the protocol of specification. The amplified products were sent to the Shanghai Biotechnology Company for sequencing and individual sequences were compared to sequences on the NCBI website to create a phylogenetic evolutionary tree and identify the bacterial species (TVBOT: https://www.chiplot.online/tvbot.html, accessed on 25 March 2024) [19].

### 2.3. Detection of DON Degradation Ability

The experimental concentration of vomitoxin was determined by high performance liquid chromatography (HPLC). Following a 48 h reaction of the MY-1 bacterial culture medium with 1 μg/mL vomitoxin at 37 °C, the supernatant was carefully removed and centrifuged at 12,000× *g* for 10 min for a second supernatant extraction. This second supernatant extract was filtered through a vomitoxin affinity purification column (HUAAN MAGNECH Bio-tech, Beijing, China) to purify the toxin. Then, the purified products were analyzed by chromatography. The chromatography analysis used a Waters XBridge C18 (5 µm 4.6 × 250 mm) column, a UV detector, and the isocratic elution technique. The experiment employed a methanol/water (20/80, VV) mobile phase ratio at a 0.8 mL/min flow rate; the column temperature was maintained at 35 °C, and 50 μL of the sample was injected. The analysis ran for 10 min and samples were detected at a wavelength of 218 nm.

### 2.4. MY-1 Strain Active Substances for Degrading DON

Strain MY-1 was inoculated into MRS liquid medium at a 1% inoculum concentration and incubated anaerobically at 37 °C for 12 h. The supernatant was obtained by centrifugation at 12,000× *g* and the precipitate was washed three times with sterile PBS buffer. An equal amount of sterile PBS buffer was used to re-suspend the precipitate, creating a cell suspension. Subsequently, the cell suspension underwent ultrasonic treatment to obtain the cell lysate. To the reaction, 990 μL of active substance and 10 μL DON (100 μg/mL) were added to maintain the DON level at 1 μg/mL. The mixture was allowed to stand at 37 °C for 48 h. Following this, the system’s DON concentration was ascertained through HPLC.

### 2.5. Animal Experimental Design

Thirty male Kunming mice, each 20 days old, were randomly segregated into two groups, i.e., the control group and the MY-1 group, with 15 mice in each group. A week prior to the experiment’s commencement, mice underwent acclimatization, with enough food and water to remain nourished, at 25 °C over a period of 28 days. The control group was given 0.2 mL of PBS per day via gavage, whereas the experimental group was given a 1 × 10^8^ CFU/mL MY-1 bacterial solution per day via gavage. The mice’s health status was monitored and their weights were taken daily. On the 29th day of the study, blood was extracted from the posterior orbital venous plexus and left to stand for two hours, before being centrifuged at 3000× *g* for 10 min at 4 °C. The serum was then collected and stored at −20 °C. Mice underwent euthanasia through cervical dislocation, followed by the computation of post-mortem organ indices for the hearts, livers, spleens, lungs, and kidneys, using the following formula:

organ index (%) = organ mass (g)/mouse body mass (g) × 100%(1)

After weighing the organs, molecular and histopathological samples were taken. Molecular samples were preserved at −80 °C, while the fixed samples were kept at 4 °C.

### 2.6. Detection of Serum Antioxidant Indicators

The serum antioxidant properties of the mice were assessed using the Jiancheng kits (Nanjing, China) in strict accordance with the manufacturer’s instructions. Malondialdehyde (MDA), glutathione peroxidase (GSH-Px) and total superoxide dismutase (T-SOD) were used as serum antioxidant markers.

### 2.7. Histopathology

Paraffin sections of all organs were routinely prepared, HE stained, sectioned, and then examined microscopically.

### 2.8. Quantitative Real-Time PCR

Total mouse RNA was extracted from organ samples using the Accurate Biotechnology RNA extraction kit (Hunan, China) following the instruction manual. Then, the RNA concentration and OD260/OD280 ratio were determined and RT-qPCR amplification was performed using cDNA templates according to the kit’s specific spiking and reaction system settings.

### 2.9. Analysis of Gut Microbial Diversity

Five mice were selected randomly from each group to collect gut contents for high-throughput sequencing. DNA samples were quality assessed and spliced to obtain high-quality sequences for OTU and species annotation analyses. Alpha and beta diversity analyses were performed for flora richness and evenness based on known OTU clustering.

### 2.10. Genome Sequence Determination and Assembly of Strain MY-1

The MY-1 strain, frozen at −20 °C, was inoculated onto the MRS solid medium for activation culture and passaged 2–3 times to ensure purity. Then, the DNA was extracted as shown in Section 2.2. This DNA was preserved at −20 °C for subsequent application.

Sangon Biotech Co., Ltd. conducted the complete genome sequencing of MY-1. (Location: Shanghai, China) on the Illumina system. The sequencing raw data underwent data statistics and quality assessment in FastQC 0.11.2 [20]. Quality clipping was also performed in order to obtain relatively accurate and valid data. The second-generation sequencing data were spliced using SPAdes 3.5.0 [21]. To enhance the spliced contigs with GAP, GapFiller 1.11 [22] was employed, while PrInSeS-G 1.0.0 [23] served to correct sequences by fixing editing errors and adding and removing small fragments during the splicing. Gene elements CDS, tRNA, rRNA, etc., were predicted using Prokka 1.10 [24] software.

### 2.11. Functional Annotation of the MY-1 Genome

NCBI Blast+ [25] was used to compare the gene protein sequences of the MY-1 genome with sequences in the COG database [26] to obtain its functional annotation information (e-value~1 × 10^−5^). Functional annotation details for GO were gathered from the genes’ annotations using Swissprot and TrEMBL [27]. The KAAS (KEGG Automatic Annotation Server) [28] was employed to obtain gene KEGG annotation information (e-value~1 × 10^−5^ and bitscores~60), for which annotations related to human diseases were eliminated. A comparison was made between the entire MY-1 genome and the Non-Redundant (NR) database at the NCBI to verify the closeness of the species’ transcript sequences to those of similar species and to gather the functional details of analogous sequences.

### 2.12. Prediction of Genes Encoding for CAZymes and Secondary Metabolites in MY-1

Protein sequences from the gene set were matched with the CAZy database (Carbohydrate-Active enZYmes Database) to obtain their respective carbohydrate-active enzyme annotation details (e-value~1 × 10^−5^, pident~40 and min length~50) [29].

Clusters of secondary metabolite genes were pinpointed via the antiSMASH web-based annotation platform (http://antismash.secondarymetabolites.org/, accessed on 18 January 2024).

### 2.13. Statistical Analysis

The experimental data were analyzed by one-way ANOVA and *t*-tests using SPSS 21.0, and multiple comparisons were made using Duncan’s method, plotted using GraphPad Prism (8.3.0) and Chiplot. The results of the experiments were expressed as “mean ± SEM”, with *p* < 0.05 indicating a significant difference and *p* < 0.01 indicating a highly significant difference.

## 3. Results

### 3.1. Screening of DON-Degrading Strains

A total of 108 colonies with different growth patterns were obtained from the samples by plate streaking in different solid media. Partially purified isolate cultures were inoculated into a primary screening medium containing DON, and 12 strains with the ability to degrade DON were identified. These were numbered LB01, LB02, LB03, NA05, NA06, NA07, NA08, MRS1, MRS2, MRS18, MY-1 and MC-1.

**Figure 1:**
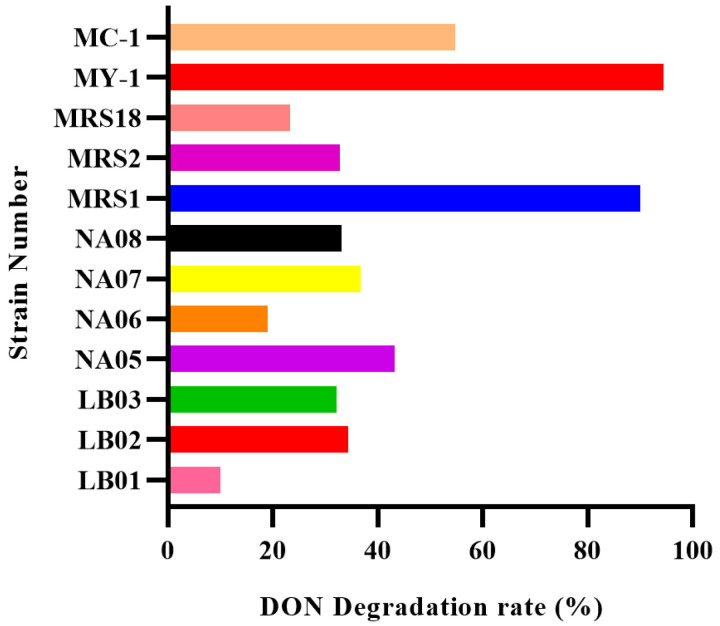
Degradation rate of DON-degrading bacterial strains.

The 12 strains obtained were then co-cultured with DON for 48 h and HPLC was used to measure the DON levels in the medium and determine the rate of degradation (Figure 1). The degradation rates of the 12 strains ranged from 10.1% to 94.53% in the MY-1 strain. 

### 3.2. L. rhamnosus Identification

**Figure 2:**
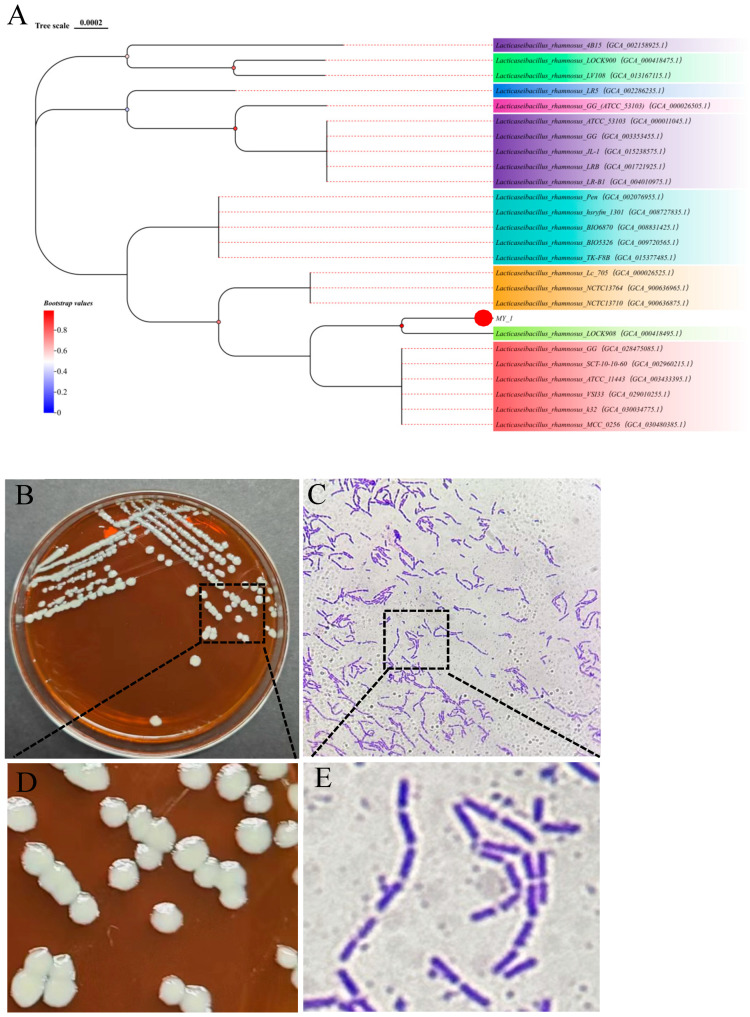
The phylogenetic tree and morphological characteristics of the DON-degrading *L. rhamnosus* MY-1 strain isolated from environmental samples in China. (**A**) Phylogenetic tree of related strains based on the 16S rRNA gene sequence. (**B**,**D**) Cultures of the MY-1 strain cultivated on MRS agar medium for 24 h at 37 °C. (**C**,**E**) Gram staining of the MY-1 strain observed through a light microscope (100×).

The 16S rRNA gene sequence comparison identified the MY-1 strain as *L. rhamnosus*. As can be observed from the phylogenetic tree in Figure 2A, *L. rhamnosus* MY-1 belongs to a branch of seven strains, including *L. rhamnosus* Lc705 and *L. rhamnosus* NCTC13764. *Lactobacillus rhamnosus* LOCK908 had the highest homology with *L. rhamnosus* MY-1, which reached 99%.

Strain MY-1 is a slow-growing Gram-positive anaerobic bacterium that often settles to form a white precipitate in liquid media. Colonies in solid media are small, creamy white, round, smooth and slightly raised (Figure 2B,D). The organisms were observed under the microscope as short bluish-violet rods, arranged singly and in chains, with no spores (Figure 2C,E).

### 3.3. DON Degradation Mechanism of MY-1 

**Figure 3:**
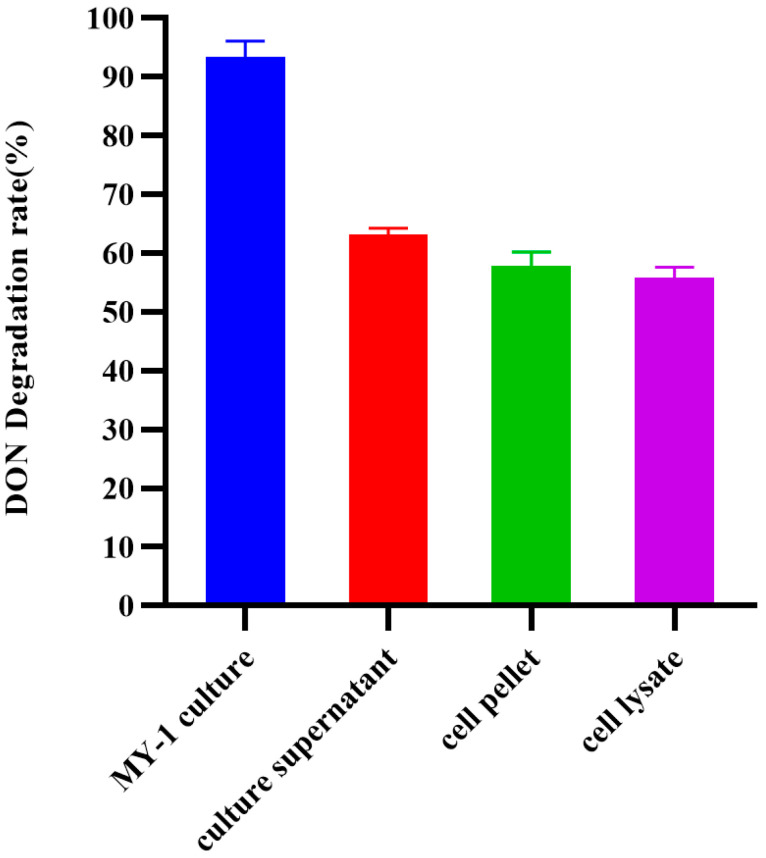
Localization of *L. rhamnosus* MY-1 strain active substances for degrading DON.

The degradation rate of DON was examined in various active substances. The degradation rate of the MY-1 culture was 93.34% and the culture supernatant degradation rate was 63.23% (Figure 3). The degradation rate of the cell pellet was 57.87% and the degradation rate of the cell lysate was 55.88%. These results suggest that *L. rhamnosus* MY-1, along with its culture supernatant and intracellular enzymes, has detoxification effects on DON and that *L. rhamnosus* MY-1 possesses both adsorption and degradation capabilities for detoxifying DON.

### 3.4. In Vivo Safety Assessment of L. rhamnosus MY-1

#### 3.4.1. Effect of *L. rhamnosus* MY-1 on Mouse Body Weight, Feed Intake and Organ Index

**Figure 4:**
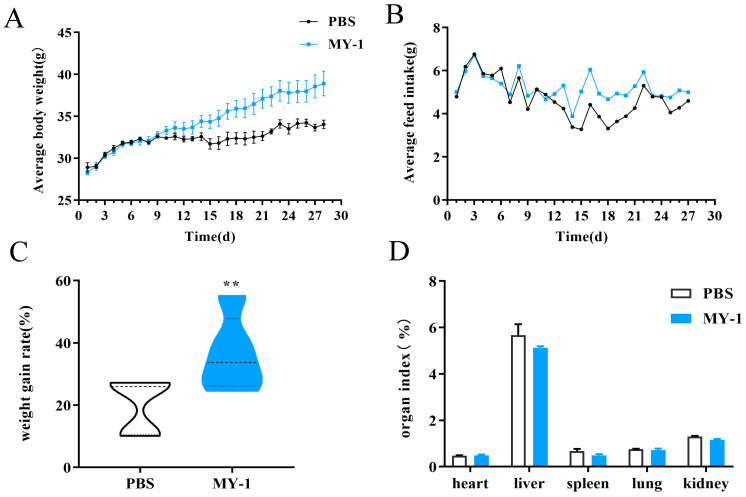
Influence of *L. rhamnosus* MY-1 on mouse body weight, feed intake and organ index. (**A**) Average body weight; (**B**) average feed intake; (**C**) weight gain rate; and (**D**) organ index. Results are shown as mean ± SEM. Significance compared to the control group: ** *p* < 0.01.

Throughout the experimental cycle, all mice had smooth coats, good mental status and health and none died during the experiment. Necropsy results showed that none of the internal organs were damaged and there were no hemorrhagic areas. The body weights of the *L. rhamnosus* MY-1 group were greater than those of the control group after day 9, with a significant increase in the rate of weight gain (Figure 4C, *p* < 0.01). The average daily feed intake also increased significantly after day 12 compared with the control group. However, there was no significant difference in organ indices between the *L. rhamnosus* MY-1 group and the control group (*p* > 0.05) (Figure 4). These findings indicate that the presence of *L. rhamnosus* MY-1 positively influenced the growth patterns of mice.

#### 3.4.2. Effect of *L. rhamnosus* MY-1 on Mouse Organ and Intestinal Tissue Morphology 

**Figure 5:**
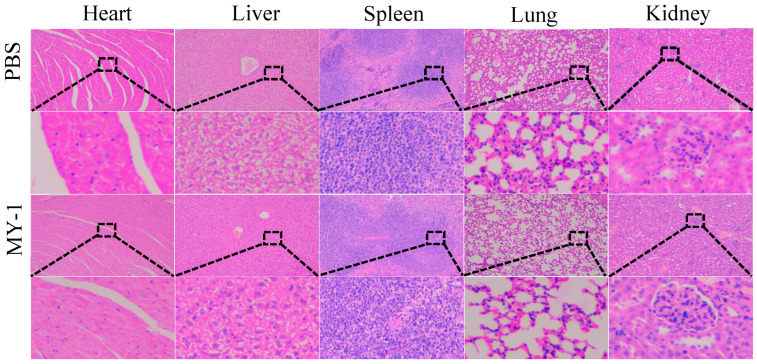
Effect of *L. rhamnosus* MY-1 exposure on mouse organ morphology. Bars = 100 μm and original magnification, 100×.

There was an absence of notable histopathological damage in the heart, liver, spleen, lungs, or kidneys of mice exposed to the *L. rhamnosus* MY-1 strain (Figure 5). The cardiomyocytes of the mouse hearts in both groups were neatly aligned, and the myocardial fibers were arranged in an orderly manner to form myocardial bundles. Liver lobules were tight and regular, with hepatocytes arranged radially around the central vein, clear interlobular structures and no obvious inflammatory reaction, necrosis or fibrosis. The spleen was clearly delineated into the splenic corpuscle, the red medullary region, the lymphoid region and the splenic sinus, and the cellular tissues were arranged in an orderly fashion. In the lungs, the alveolar tissue was complete and clear, and the alveolar and bronchial lumens were very clean. In the kidneys, the glomeruli were tightly packed, the vessel walls were intact, and the individual structures were normal.

**Figure 6:**
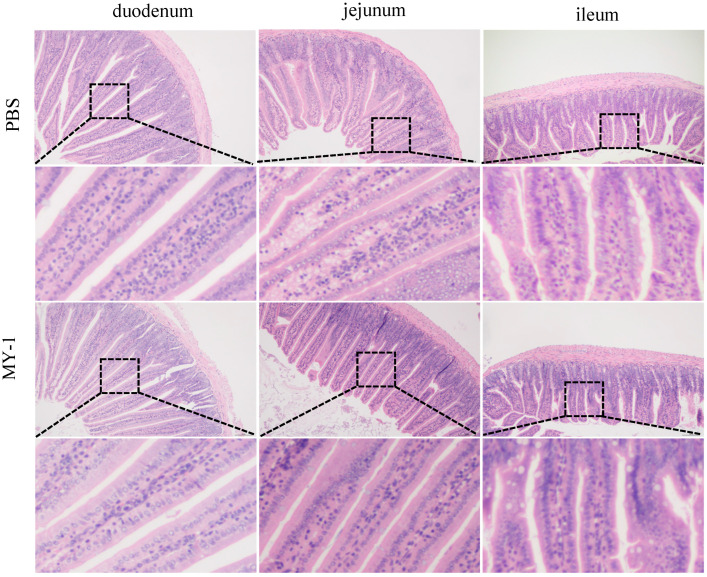
Effect of *L. rhamnosus* MY-1 on mouse intestinal tissue morphology. Bars = 100 μm and original magnification, 100×.

The morphology and structure of each segment of the small intestine in the control group and the *L. rhamnosus* MY-1 exposed group were normal, the intestinal villi were neatly and densely arranged, the morphology of the epithelial cells was normal, and the surface of the crypts was smooth with no obvious cell proliferation (Figure 6). Compared with the control group, the length of the duodenal villus, the depth of the crypt, and the VCR (the length of villus/the depth of crypt ratio) showed no notable variance in the *L. rhamnosus* MY-1-exposed group. The same was seen in the jejunum (*p* > 0.05). The length of the ileal villi in the MY-1 group was significantly greater than that in the control group (*p* < 0.01), and there was no notable variance in crypt depth and VCR (*p* > 0.05) (Table 1).

#### 3.4.3. Effect of *L. rhamnosus* MY-1 on Mouse Antioxidant Levels

To investigate the extent of oxidative stress and damage after exposure to *L. rhamnosus* MY-1, we examined the serum levels of malondialdehyde (MDA), superoxide dismutase (SOD) and glutathione peroxidase (GSH-PX) in mice.

**Figure 7:**
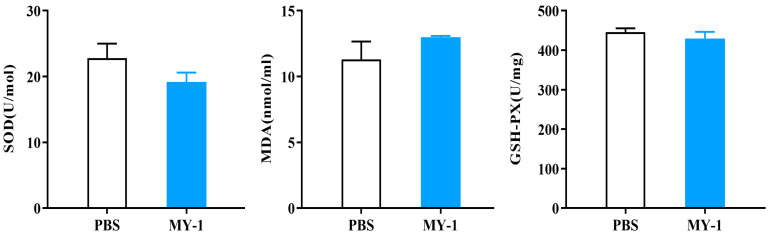
Effect of *L. rhamnosus* MY-1 on the antioxidant levels in mice. Results are shown as mean ± SEM. Significance compared to the control group.

As shown in the Figure 7, the SOD and GSH-PX levels were slightly decreased in the *L. rhamnosus* MY-1 group compared to the control group, but the difference was not significant (*p* > 0.05). The MDA content of the experimental group was slightly higher than that of the control group, but the difference was also not significant (*p* > 0.05).

#### 3.4.4. Effect of *L. rhamnosus* MY-1 on Inflammatory Cytokines in Mice

To assess the inflammatory state of the body and the extent to which *L. rhamnosus* MY-1 affected the mice, the expression of the pro-inflammatory cytokines IL-1β, TNF-α, IL-6 and IL-10 was tested.

**Figure 8:**
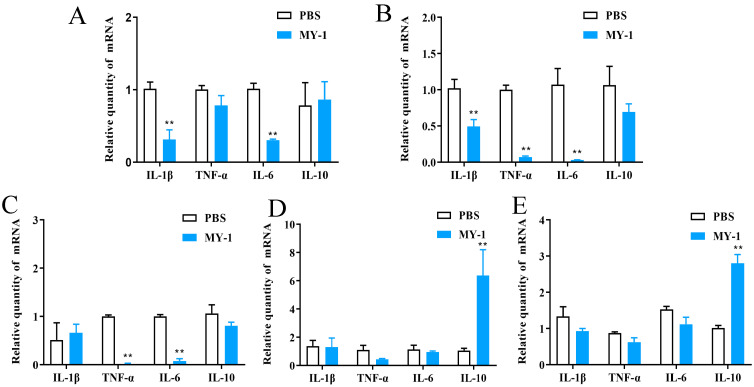
Effect of *L. rhamnosus* MY-1 on the expression of inflammatory factors in mice. (**A**) Relative expression of mRNA in the spleen; (**B**) relative expression of mRNA in the kidney; (**C**) relative expression of mRNA in the duodenum; (**D**) relative expression of mRNA in the jejunum; and (**E**) relative expression of mRNA in the ileum. Results are shown as mean ± SEM. Significance compared to the control group: ** *p* < 0.01.

In the experimental group, the levels of IL-1β, TNF-α, and IL-6 in the spleen and kidney were markedly lower compared to the control group (*p* < 0.05), with no notable variance in IL-10 expression (*p* > 0.05). In the experimental group’s duodenum, the expression of TNF-α and IL-6 were decreased compared to the control group (*p* < 0.01), with no notable variance in IL-1β and IL-10 expression (*p* > 0.05). Although the experimental group’s jejunum and ileum showed no notable variance in pro-inflammatory cytokine levels (*p* > 0.05), the expression of the anti-inflammatory agent IL-10 was markedly greater compared to the control group (*p* < 0.01) (Figure 8).

#### 3.4.5. Effect of *L. rhamnosus* MY-1 on the Microbial Diversity of the Mouse Cecum

**Figure 9:**
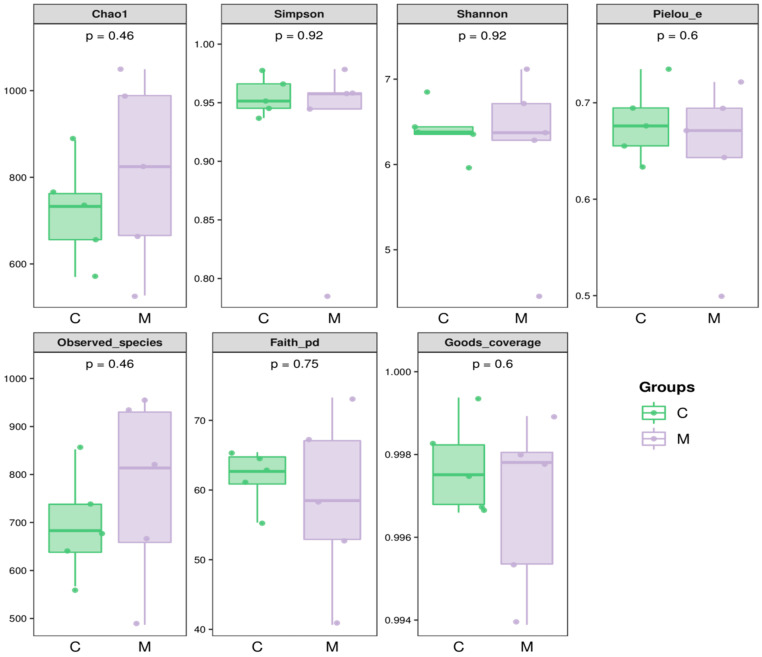
Alpha microbial diversity analysis of the gut microbiome of mice exposed to *L. rhamnosus* MY-1 (n = 5). C represents the control group, while M represents the *L. rhamnosus* MY-1 group.

To provide a more comprehensive assessment of the microbial alpha diversity in the mouse cecum, we characterized richness with the Chao1 and Observed species indices, diversity with the Shannon and Simpson indices, evolutionary diversity with the Faith’s PD index, evenness with the Pielou’s evenness index and coverage with the Good’s coverage index. The results indicate that the difference between the control group and the experimental group was not statistically significant (*p* > 0.05) (Figure 9).

Beta diversity defines the variation in species makeup or the speed at which species change across an environmental gradient among diverse communities along this gradient, thus referred to as between-habitat diversity. Multidimensional microbial data can be downscaled using unconstrained sorting methods such as Principal Coordinate Analysis (PCoA), Nonmetric Multidimensional Scaling (NMDS), and by spreading samples across successive sorting axes to reveal the prominent trends of data changes.

**Figure 10:**
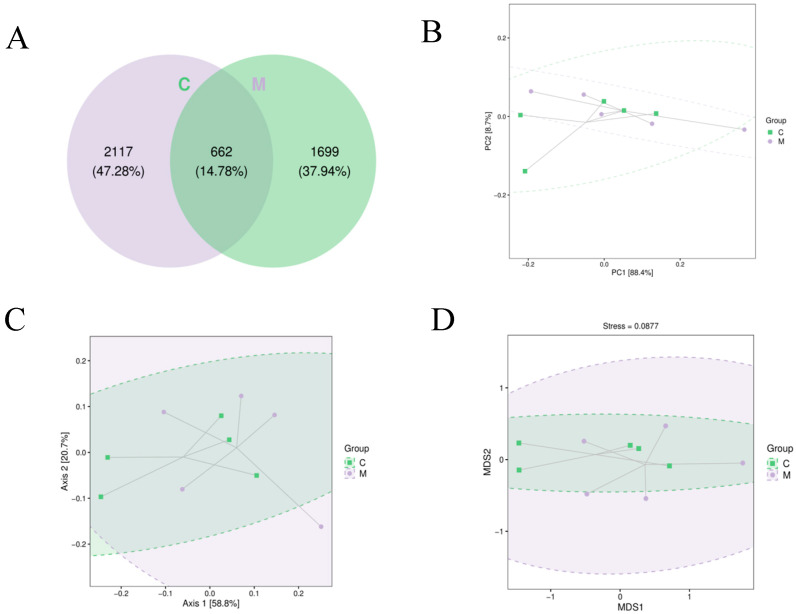
Diversity analysis of the β-gut microbiome (n = 5) of mice exposed to *L. rhamnosus* MY-1. C represents the control group, while M represents the *L. rhamnosus* MY-1 group. (**A**) A Venn diagram derived from OTU levels. (**B**) PCA analysis focusing on OTU levels; the greater the similarity in the community makeup of the samples, the nearer they appear in the PCA graph. (**C**) PCoA analysis using weighted UniFrac distance; the nearer the sample distances, the greater their resemblance in species composition. (**D**) NMDS analysis using weighted UniFrac distances; the closer the point-to-point distances in the samples, the more alike their microbial community structure.

As shown in Figure 10A, the Venn diagram indicated that the total OTUs in the control and experimental groups were 14.78%. Control-group-specific OTUs accounted for 47.28%, while experimental-group-specific OTUs accounted for 37.94%. Findings of the β-diversity analysis were showcased through PCA, PCoA, and NMDS downscaling techniques (Figure 10B–D). The PCA analysis was performed based on OTU levels, while the PCA clustered all samples within a range except for one outlier. The PCoA and NMDS analyses were performed based on the UniFrac evolutionary distance to assess the similarity between samples. In the PCoA analysis, all samples, except one significant outlier, clustered within a range. In the NMDS analysis, the stress was equal to 0.0877 (<0.2) based on the UniFrac evolutionary distance. All species diversity analyses showed no notable variance between the experimental and control groups.

**Figure 11:**
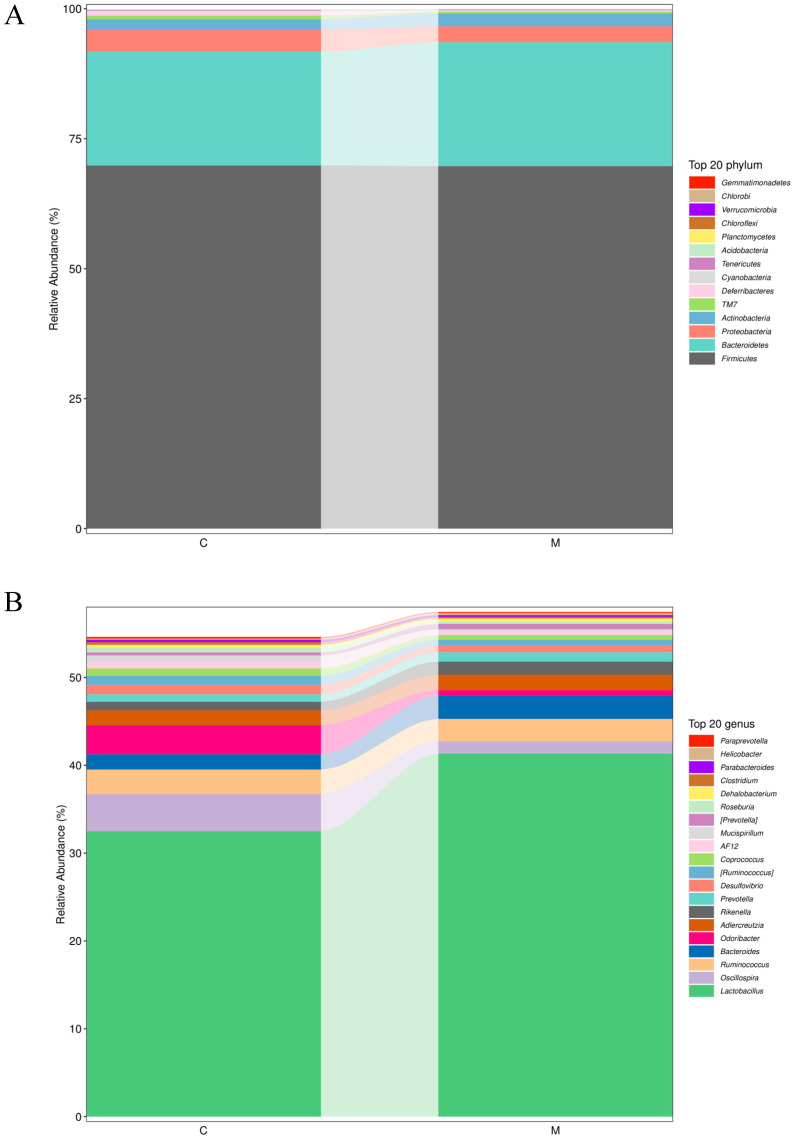
The makeup of gut microbial communities in mice exposed to *L. rhamnosus* MY-1 and the prevalent taxa within these groups (n = 5). C represents the control group, while M represents the *L. rhamnosus* MY-1 group. Relative abundance of major microbiomes at the (**A**) phylum level and (**B**) genus level.

At the phylum level (Figure 11A), a selection of the top 20 phyla was made to create a columnar cumulative graph depicting their comparative abundance. The Firmicutes, Bacteroidetes, Proteobacteria and Actinobacteria phyla dominated the intestinal microbiota, with the proportion of these four phyla collectively exceeding 95%. Compared with the control group, there was no significant difference in the abundance of the dominant phyla in the MY-1 group (*p* > 0.05). At the genus level (Figure 11B), the six genera with the highest abundance were *Lactobacillus*, *Oscillospira*, *Ruminococcus*, *Bacteroides*, *Odoribacter* and *Adlercreutzia.* The abundance of *Lactobacillus* and *Bacteroides* in the MY-1 group surpassed that of the control group, yet the difference was not statistically significant (*p* > 0.05). In terms of *Oscillospira* and *Odoribacter* abundance, the MY-1 group’s numbers were lower than the control group’s, but the difference was not statistically significant (*p* > 0.05).

### 3.5. Lactobacillus rhamnosus MY-1 Basic Genome Information

**Figure 12:**
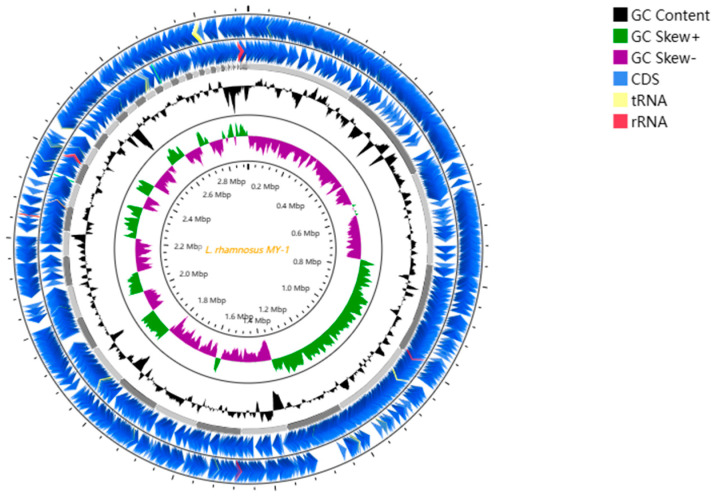
Complete genome map of *L. rhamnosus* MY-1. Circles are marked from the exterior to the interior: circles 1 and 2 (blue) signify the forward and reverse strands, symbolizing genes associated with CDS, tRNA and rRNA. The third circle, marked in black, represents the genome’s GC percentage. The fourth circle, colored in purple and green, symbolizes a skew in the GC content.

Table 2 and Figure 12 display the genetic features of *L. rhamnosus* MY-1. The total genome length was 2,904,588 bp, and the total length of the coding genome was 2,445,741 bp with 2706 coding genes accounting for 84.203% of the total length. The G + C content was 47% and the genome contained 55 tRNAs, 6 rRNAs and 1 ncRNA. The Whole Genome Shotgun Initiative is stored in GenBank, accessible under the identifier JBCARB000000000.

### 3.6. Protein Function Prediction of L. rhamnosus MY-1

**Figure 13:**
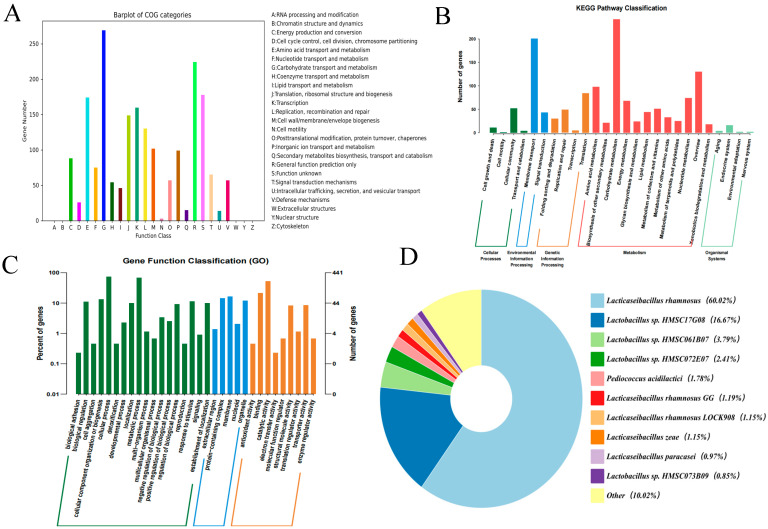
Protein prediction function of *L. rhamnosus* MY-1. (**A**) COG categories of functional proteins; (**B**) GO annotation results; (**C**) KEGG pathway enrichment; and (**D**) a pie chart depicting the homology distribution of *L. rhamnosus* MY-1.

Every gene coding for proteins was marked using the protein direct homology cluster database (COG). As shown in Figure 13A, the 1844 coding protein genes in the MY-1 genome were grouped into 20 COG subclasses, accounting for 68.14% of all coding protein genes. The *L. rhamnosus MY-1* strain contains numerous genes involved in carbohydrate transport and metabolism and general function prediction only, followed by amino acid transport and metabolism, transcription and function unknown.

Genetic sequences of the proteins produced by *L. rhamnosus* MY-1 were matched with the Gene Ontology (GO) database to gather functional annotation details. As shown in Figure 13B, a total of 441 genes were enriched in 32 GO terms, which were classified into Biological Processes and Cellular Component and Molecular Function. The largest number of genes enriched in the Biological Processes category were those involved in cellular and metabolic processes. The largest number of genes in the Cellular Components category were related to protein-containing complex, as well as membrane and organelle. The most annotated genes in the broad category of molecular functions were related to catalytic activity and binding, followed by transporter activity and structural molecule activity. This is an indication that *L. rhamnosus MY-1* may be highly metabolically active, with significant signaling capacities and a high protein content.

Comparing the whole genome of *L. rhamnosus* MY-1 with the KEGG database, 833 genes encoding proteins were enriched in five major pathway categories: cellular processes, metabolism, genetic information processing, organic systems and environmental information processing. The majority of genes across all KEGG pathways play a role in the metabolism of carbohydrates and movement of membranes, suggesting a metabolically active strain (Figure 13C).

A pie chart depicting the distribution of MY-1 homology was created by contrasting the genome of the *L. rhamnosus* MY-1 strain with the NR database. Every segment in the chart symbolizes a different species, and the more extensive the sector’s size, the higher the count of sequences relative to that species. Figure 13D illustrates that strain MY-1 shares a high degree of homology with *Lactobacillus rhamnosus* spp.

### 3.7. CAZy Database Annotation Result and Prediction of Secondary Metabolites of L. rhamnosus MY-1

CAZy is a specialized database of carbohydrate-active enzymes, including related enzyme families that catalyze carbohydrate degradation, modification and biosynthesis.

Among them, the *L. rhamnosus MY-1* genome identified 70 carbohydrate enzymes, including 38 GH, 19 GT, 12 CE and 1 AA. The genes of glycoside hydrolases and glycosyl transferases topped the list, representing 54.3% and 27.1% of all the CAZymes genes, in that order (Table 3).

**Figure 14:**
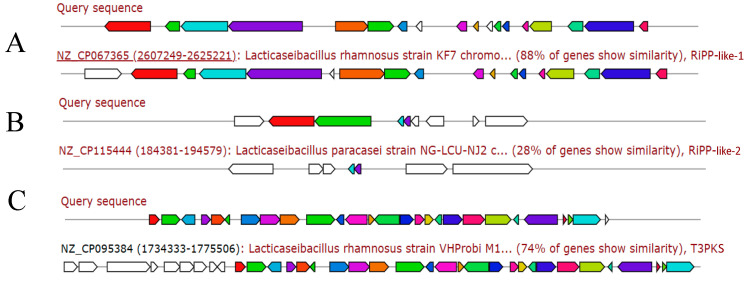
Analysis of secondary metabolites of *L. rhamnosus* MY-1: (**A**) RiPP-like-1; (**B**) RiPP-like-2; and (**C**) T3PKS.

Comparison of the whole-genome sequence of MY-1 with the secondary metabolite database led to the identification of two gene clusters encoding secondary metabolites with antimicrobial properties in the *L. rhamnosus MY-1* genome (Figure 14). There were two clusters of genes encoding RiPP-like (ribosomally synthesized and post-translationally modified peptide-like), one of which was involved in the synthesis of RiPP-like with 88% similarity. There was also a cluster of genes encoding T3PKS (class III polyketide synthase) with 74% similarity to *Lactobacillus rhamnosus* NZ_CP095384.1.

## 4. Discussion

Recent surveys have shown that crops are predominantly contaminated with *Fusarium* mycotoxins globally, with DON contamination being the most widespread and dangerous [30]. Therefore, the prevention and control of DON contamination urgently requires a new technology with high specificity, high efficiency, no feed damage and environmental benefits [31]. Microbial biological detoxification is efficient, safe and environmentally friendly and is an important breakthrough and directive in DON degradation research [10].

In this study, a Gram-positive bacterial strain, *Lactobacillus rhamnosus* MY-1, with efficient DON-degrading ability was screened from moldy straw and initially identified by 16S rRNA sequencing. The *L. rhamnosus* MY-1 degradation mechanism of DON involves the synergistic effect of bioadsorption and biodegradation. This dual action underscores its promising potential as a feed detoxifier.

*Lactobacillus rhamnosus* MY-1, isolated in this study, effectively mitigated DON, achieving an impressive degradation rate of 93.34%. At present, the most common DON-degrading bacteria belong to the *Devosia* and *Bacillus* genera, with degradation rates of more than 80% in *Devosia*. The degradation rate of *Devosia* A6, isolated by Wang et al., reached 80% at 35 °C [32], while *Devosia* 17-2-E-8 and *Devosia* D6-9 can degrade DON sustainably in a bacterial fermentation solution [33,34]. The degradation rate of *Bacillus* ranges from 66% to 92%, and all toxins are effectively degraded by the bacterial fermentation fluid [35,36]. Therefore, the degradation rate of *L. rhamnosus* MY-1 is very advantageous. Most of the bacteria reported in the *Lactobacillus* genus degrade DON by adsorption [9], and the biodegradation rate of the *L. rhamnosus* supernatant was 63.32%. *Lactobacillus rhamnosus* RC007, isolated by Garcia et al., was used to degrade DON by a supernatant, but the degradation rate was only 13% [37], while the *L. rhamnosus* SHA113, isolated from breast milk by Qu et al., can convert DON to 3-epi-DON, and the degradation rate can reach 60% within 24 h [38]. Therefore, further investigation is warranted to explore the enzymatic action that may contribute to the high degradation rate of this *Lactobacillus rhamnosus* MY-1 supernatant.

After exploring the detoxification ability of *L. rhamnosus* MY-1 in vitro, we then investigated the safety and benefit of *L. rhamnosus* MY-1 in animals. The safety and beneficial properties of *Lactobacillus rhamnosus* has been studied before [39,40,41], and focused on animal testing [42,43]. Twenty-day-old mice, which were used in this study, are still developing and possess a nascent intestinal epithelial barrier and are thus vulnerable to infection and apt for application in toxicological modeling [44]. After 28 days of chronic toxicity testing, the mice in both groups had smooth fur, good mental status and good health. The mice in the experimental group exhibited a notably greater body weight compared to the control group, and their feed consumption exceeded that of the control group, aligning with Bogsan et al.’s findings [45]. This suggests that *L. rhamnosus* MY-1 is beneficial for increasing dietary intake in mice, improving animal performance, and is suitable for use in feed as a feed additive. In addition, HE staining of the internal organs showed no pathological changes in any of the tissues, in agreement with the findings of Li et al. [44]. This suggests that *L. rhamnosus MY-1* has no toxic effect on mouse organs in vivo.

Superoxide dismutase (SOD) is an important antioxidant enzyme, MDA (malondialdehyde) is a product of intracellular lipid peroxidation, and GSH-PX (glutathione peroxidase) is an enzyme involved in antioxidant defense along with glutathione [46]. All three indicators are related to the oxidative state of cells and oxidative damage [47]. The experimental and control groups showed no notable disparity, suggesting that MY-1 does not adversely impact the organism’s antioxidant abilities, a finding supported by prior research findings [48]. Inflammatory cytokine expression levels are commonly used as an indicator of the degree of inflammation and this work found a notable decrease in pro-inflammatory elements in the spleen, kidney and duodenum, alongside a substantial rise in anti-inflammatory elements in the jejunum and ileum of the test group, implying that *L. rhamnosus* MY-1 controls inflammatory factor expression and diminishes inflammation risk in mice. Similar results were reported by Qiu et al. [49], increasing the credibility of our experiment.

The gut is a complex ecosystem [50], where variations in the length of the small intestinal villi, the depth of the crypts, and V/C ratios serve as crucial measures for the digestive system’s nutrient absorption and digestive capabilities, and for evaluating the intestinal barrier’s health [51]. In the *L. rhamnosus* MY-1 group, the villi and crypts of the small intestine remained intact, and there was a significant increase in the length of the ileum villi. Previous research has demonstrated that probiotic supplementation can enhance ileum villi length and the V/C ratio, as well as preserve ileum villi integrity [52]. These findings are consistent with our own, supporting the notion that supplementation with *L. rhamnosus* MY-1 supports the maintenance of intestinal morphology in mice. The primary roles of gut microbiota encompass serving as an immune shield for the intestinal lining, assisting in food digestion and absorption, and enhancing nutrient processing [53], and the normal physiological functions of the host can be negatively affected due to changes in the gut microbiota. Our results showed that there was no notable variance in the gut flora between the experimental group and the control group, indicating that the addition of *L. rhamnosus* MY-1 does not affect the abundance or composition of the gut flora in the cecum of mice and does not cause any adverse effects in mice.

Finally, we explored the functional genes of MY-1 by whole-genome sequencing and bioinformatic analysis. The whole genome size was determined to be 2,904,588 bp, with a total of 2706 coding genes, accounting for 84.203% of the full length. The G + C content was 47%, similar to the *Lactobacillus rhamnosus* genome 10 studied by Zhao et al. [39]. The genomic data were compared and analyzed with GO, COG, KEGG, NR and other databases to complete the functional annotation of the MY-1 strain genome and data statistics. Basic gene function annotation indicates that most of the *L. rhamnosus* MY-1 genes are involved in carbohydrate metabolism and membrane translocation [54].

Additionally, we forecasted the carbohydrate content in strain MY-1 and discovered that GHs and GTs possessed the greatest gene count, representing 54.3% and 27.1% of all the CAZymes genes, respectively. The presence of GTs is crucial for the development of surface structures that can be recognized by the immune system [55]. The MY-1 strain is rich in GHs and GTs, suggesting its potential as a probiotic for combating pathogens and immune stimuli, thereby enhancing mammalian immunity. The efficacy of antibiotics is a crucial factor in choosing novel probiotic varieties, given their ability to preserve gut balance by hindering the proliferation of gut pathogens [56]. In this study, two clusters of secondary metabolite genes encoding antimicrobial properties were identified in the *L. rhamnosus* MY-1 genome. It contains two clusters of genes encoding RiPP-like (ribosomally synthesized and post-translationally modified peptide-like). Unlike conventional RiPP, RiPP-like does not rely exclusively on ribosomal synthesis, but can also involve other non-ribosomal pathways. For applications in natural product discovery and biotechnology, RiPP and RiPP-like gene clusters have important potential as they produce compounds with antibacterial, antitumor, and anti-inflammatory activities [57]. Intensive study of the RiPP and RiPP-like gene clusters could help in the discovery of new drugs and bioactive substances. This study suggests that *L. rhamnosus* MY-1 produces antimicrobial compounds that help the body to fight infections caused by pathogens.

## 5. Conclusions

A strain of *L. rhamnosus* MY-1 with a high DON degradation rate, excellent safety performance and a well-defined genetic background was isolated from moldy straw. The combination of bacterial adsorption and biodegradation resulted in a degradation rate of 93.34% of DON after 48 h. Animal experiments demonstrated that *L. rhamnosus* MY-1 had no adverse effects on feeding, weight gain, tissue morphology, the antioxidant index, inflammatory factor expression, intestinal tissue integrity or the intestinal physical barrier in mice, indicating its high safety profile. Whole genome analysis revealed a clear genetic background for MY-1 along with a plethora of functional genes responsible for producing carbohydrate enzymes with antimicrobial properties and encoding secondary metabolites with antimicrobial effects.

## Figures and Tables

**Table 1:** Intestinal villus length, crypt depth and V/C values of mice exposed to *L. rhamnosus* MY-1; data are presented as mean ± SEM deviation (n = 3), ** *p* < 0.01. CON represents the control group.

		CON	*L. rhamnosus* MY-1
Duodenum	Villus height, µm	645.65 ± 39.89	581.96 ± 14.18
	Crypt depth, µm	151.38 ± 26.59	142.39 ± 13.99
	VCR	4.34 ± 0.83	4.11 ± 0.51
Jejunum	Villus height, µm	435.76 ± 19.73	435.72 ± 30.31
	Crypt depth, µm	120.67 ± 25.82	101.96 ± 17.86
	VCR	3.71 ± 0.74	4.35 ± 0.76
Ileum	Villus height, µm	212.26 ± 13.55	262.42 ± 6.48 **
	Crypt depth, µm	85.73 ± 10.93	96.02 ± 5.32
	VCR	2.49 ± 0.25	2.74 ± 0.09

**Table 2:** General genome features of *L. rhamnosus* MY-1.

Class	Number
Size (base)	2,904,588
G + C content (%)	47
Protein Coding Genes	2706
Contig num	59
Min length (base)	5077
Max length (base)	272,377
Average length (base)	49,230.31
Total coding gene (base)	2,445,741
Coding ratio (%)	84.203
tRNA	55
rRNA	6
ncRNA	1

**Table 3:** Distribution of the carbohydrate-active enzyme (CAZy) family proteins identified in the genome of *L. rhamnosus* MY-1.

Carbohydrate-Active Enzyme Family	Number of Genes	Gene Subfamily (Number of Genes)
Glycoside hydrolases, GHs	38	GH1(3), GH2(1), GH8(1), GH13(9), GH8(1), GH25(2), GH28(1), GH29(3), GH30(1), GH31(1), GH32(1), GH35(1), GH36(3), GH43(1), GH59(1), GH65(1), GH73(1), GH78(1), GH88(1), GH115(1)
Glycosyl transferases, GTs	19	GT2(10), GT4(3), GT5(1), GT8(3), GT28(1), GT35(1)
Carbohydrate esterases, CEs	12	CE1(5), CE3(1), CE7(1), CE9(2), CE10(3)
Auxiliary activities, AAs	1	AA1(1)

## Data Availability

The original contributions presented in the study are included in the article, further inquiries can be directed to the corresponding author.
